# Supplementary material for: Single cell RNA sequencing identifies early diversity of sensory neurons forming via bi-potential intermediates
Source: Nat Commun. 2020 Aug 21;11:4175. doi: 10.1038/s41467-020-17929-4 (PMC7442800; doi:10.1038/s41467-020-17929-4)
Supplement: Supplementary file 1 — Supplementary Information [file 41467_2020_17929_MOESM1_ESM.pdf]

# Single cell RNA sequencing identifies early diversity of sensory neurons forming via bi-potential intermediates

Faure, L. *et al*.

## Supplementary Figures

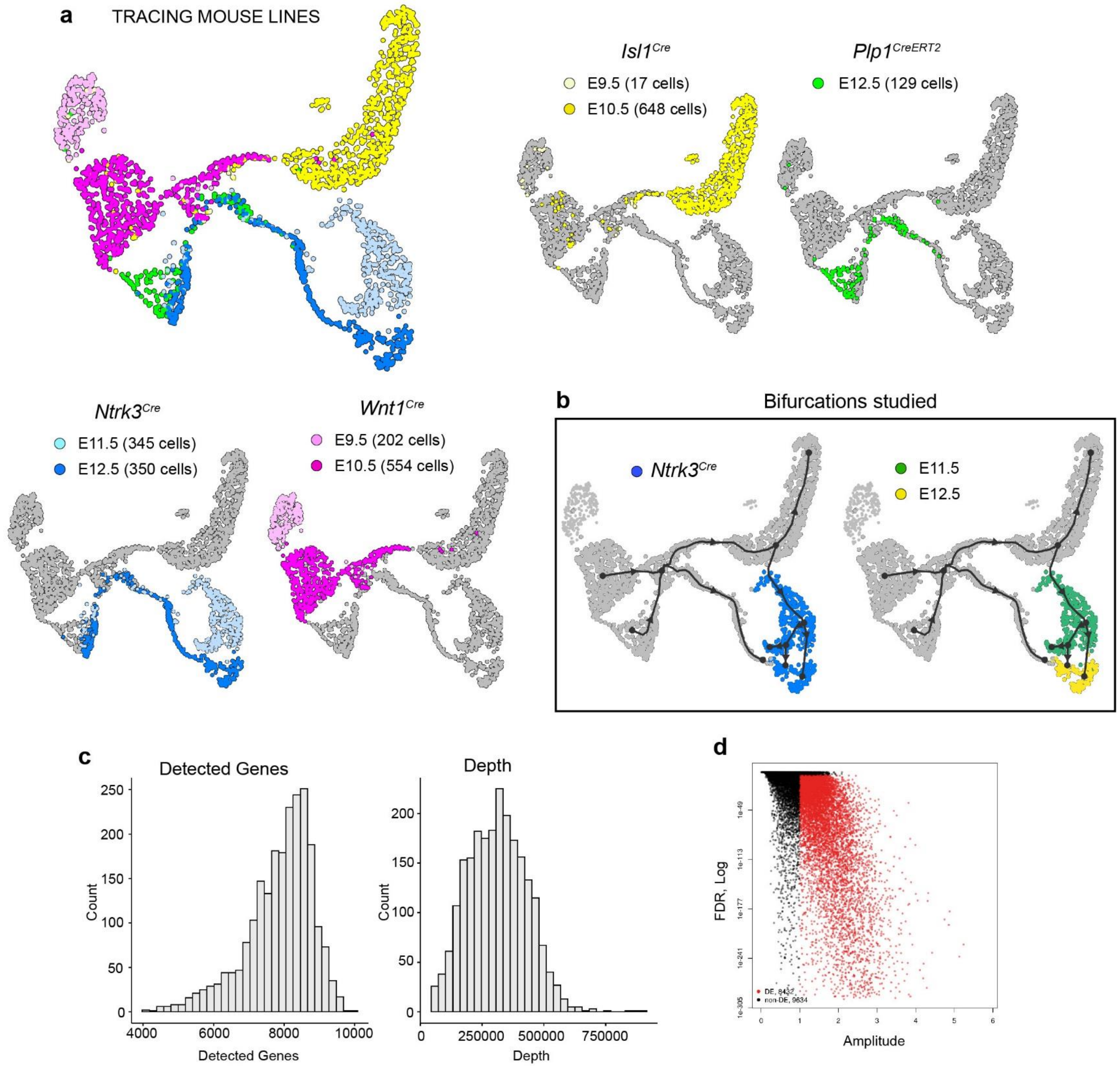


**Supplementary Figure 1. UMAPs and sequencing data quality.** (a) UMAP with color code that correspond to the conditional mouse lines used in the study with corresponding numbers of traced cells. (b) Bifurcation studied correspond to cells of the *Ntrk3* lineage (c) Number of detected genes and depth of the sequencing data. (d) Differentially expressed genes in the dataset. (Related to Figure 1).

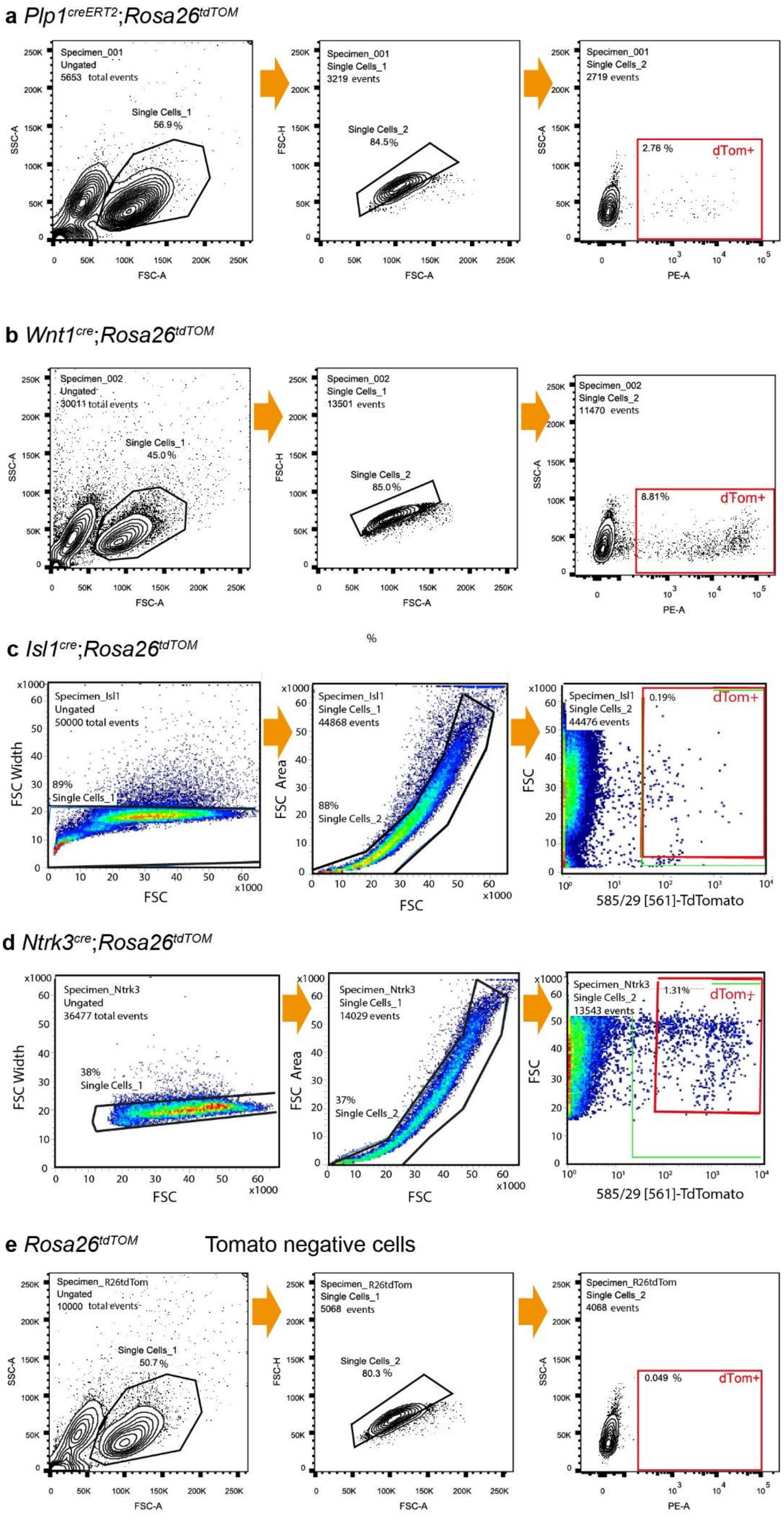

a Plp1creERT2;Rosa26tdTOM
Specimen_001
Ungated
5653 total events
Single Cells_1
56.9 %
SSC-A
FSC-A
Specimen_001
Single Cells_1
3219 events
Single Cells_2
84.5%
FSC-H
FSC-A
Specimen_001
Single Cells_2
2719 events
2.76 %
dTom+
SSC-A
PE-A
b Wnt1cre;Rosa26tdTOM
Specimen_002
Ungated
30011 total events
Single Cells_1
45.0 %
SSC-A
FSC-A
Specimen_002
Single Cells_1
13501 events
Single Cells_2
85.0%
FSC-H
FSC-A
Specimen_002
Single Cells_2
11470 events
8.81%
dTom+
SSC-A
PE-A
%
c Isl1cre;Rosa26tdTOM
Specimen_Isl1
Ungated
50000 total events
89%
Single Cells_1
FSC Width
FSC
Specimen_Isl1
Single Cells_1
44868 events
88%
Single Cells_2
FSC Area
FSC
Specimen_Isl1
Single Cells_2
44476 events
0.19%
dTom+
FSC
585/29 [561]-TdTomato
d Ntrk3cre;Rosa26tdTOM
Specimen_Ntrk3
Ungated
36477 total events
38%
Single Cells_1
FSC Width
FSC
Specimen_Ntrk3
Single Cells_1
14029 events
37%
Single Cells_2
FSC Area
FSC
Specimen_Ntrk3
Single Cells_2
13543 events
1.31%
dTom+
FSC
585/29 [561]-TdTomato
e Rosa26tdTOM
Tomato negative cells
Specimen_R26tdTom
Ungated
10000 total events
Single Cells_1
50.7 %
SSC-A
FSC-A
Specimen_R26tdTom
Single Cells_1
5068 events
Single Cells_2
80.3 %
FSC-H
FSC-A
Specimen_R26tdTom
Single Cells_2
4068 events
0.049 %
dTom+
SSC-A
PE-A

**Supplementary Figure 2. Gating strategy for each mouse lines.** For each mouse line the plots progression from left to right show the strategy used to first gated out debris and erythrocytes from the total events using FSC-A versus SSC-A plotting. Doublets were further gated out using FSC-A versus FSC-H plotting. Refinement of the single cells was performed by plotting FSC Area versus FSC. Finally, FSC-A versus PE-A (corresponding to TOMATO+ signal) to select cells that were TOMATO+ (**a**, **b** and **e**) or we plotted the FSC versus the 585/29 [561]-tdtomato (corresponding to TOMATO+ signal, **c**,**d**). A cell preparation negative for TOMATO was used to define the negative population (**e**). Note, the BD Influx instrument was manually set up and calibrated on daily basis using BD CST and BD FACS Accudrop beads. (Related to Figure 1).

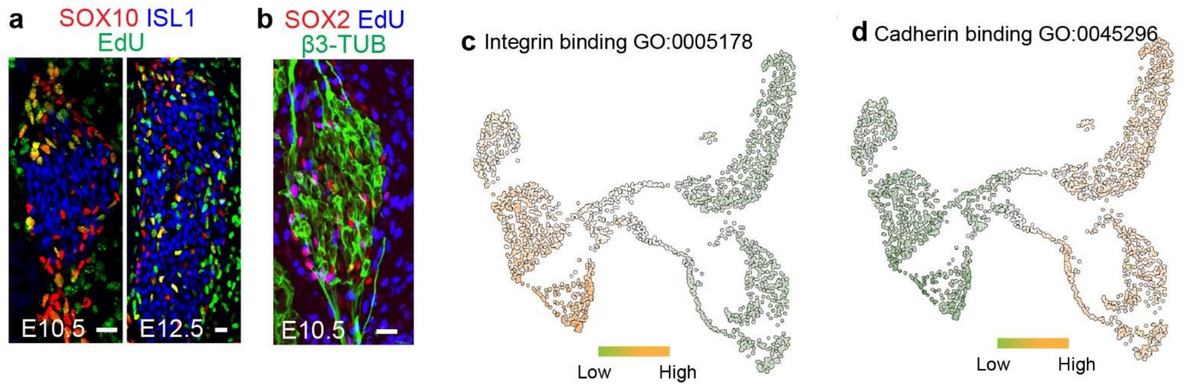


**Supplementary Figure 3. Validation of cycling versus postmitotic markers expression *in vivo* and cell migration, cell adhesion on UMAP**. (**a**,**b**) Immunostaining of SOX2, β3-TUB, SOX10 and ISL1 on E10.5 and E12.5 DRG sections from embryos of mother injected with EdU (2h pulse), showing distinct pool of SOX10+ cells that do not co-localize with postmitotic neuronal marker ISL1 while co-localize with the cell cycling marker EdU (n=3). Scale bars, 20µm. (**c**) UMAP displaying Integrin binding GO:0005178 . It shows that integrin and integrin binding molecules are highly expressed in the stem cell pools while down-regulated in the rest of the tree. (**d**) UMAP displaying cadherin binding GO:0045296, showing opposite distribution that the integrin in (**c**). (Related to Figure 1).

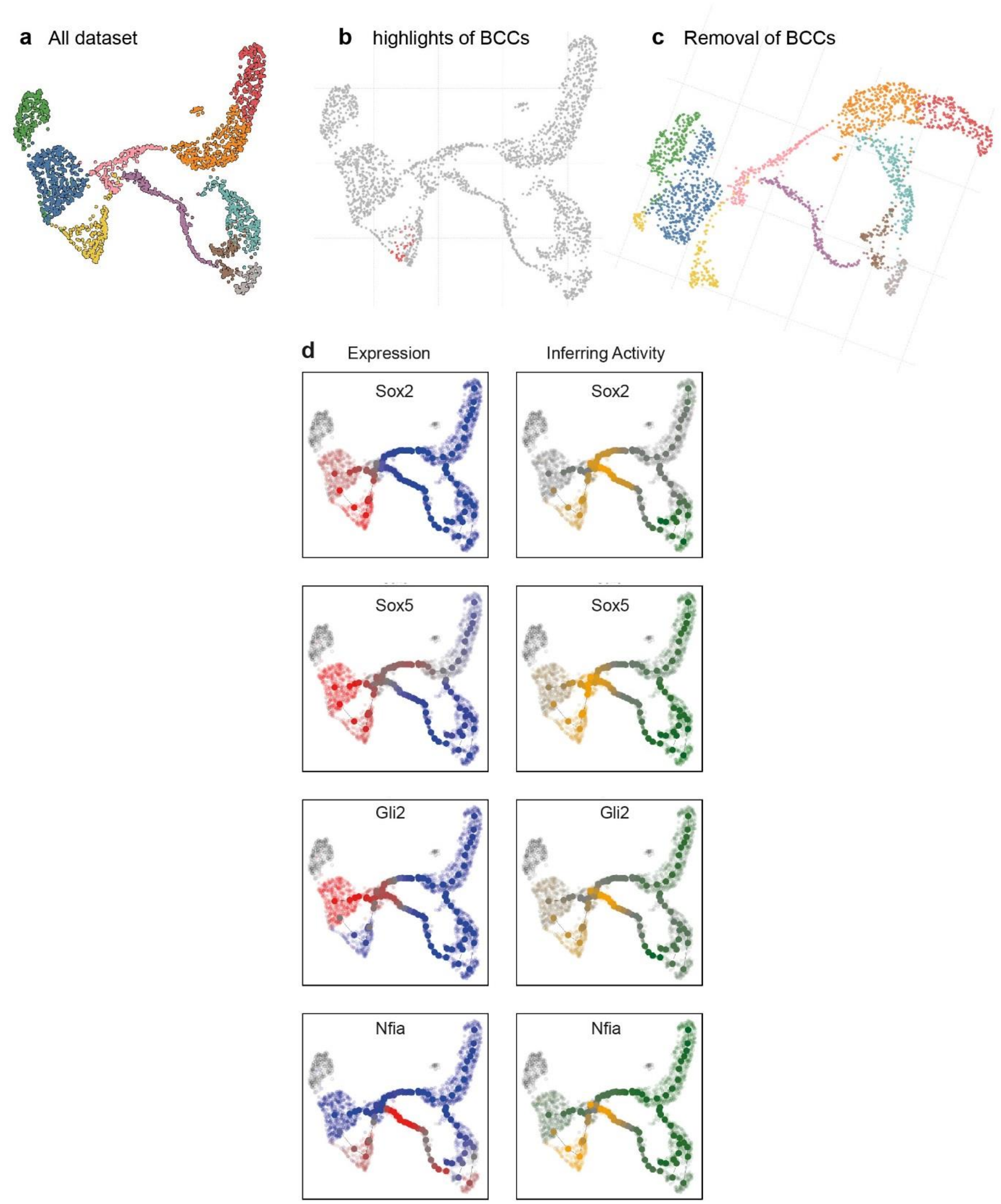


**Supplementary Figure 4. UMAP embedding representation of the single cell RNA sequencing dataset. a**, UMAP embedding representation of the single cell RNA sequencing with the all data set or after BCCs removal in **c**. **b**, Highlight of BCCs in red. **d**, Transcription factors expression pattern (left panel) and inferring activity (right panel) of those TFs along the trajectories. (Related to Figure 2).

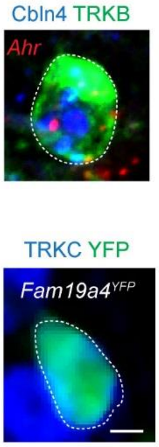


**Supplementary Figure 5. Immunostaining and in-situ hybridization on DRG sections confirm expression of potential markers**. Marker of mechanoreceptor Cbln4 is co-localizing in TRKB cells (top panel) and marker of proprioceptors (Fam19a4) co-localizing with TRKC+ cells (bottom panel) at E12.5 (n=3). Scale bar: 10µm. (Related to Figure 3).
