# Supplementary material for: Single cell RNA sequencing identifies early diversity of sensory neurons forming via bi-potential intermediates
Source: Nat Commun. 2020 Aug 21;11:4175. doi: 10.1038/s41467-020-17929-4 (PMC7442800; doi:10.1038/s41467-020-17929-4)
Supplement: Supplementary file 2 — Reporting Summary [file 41467_2020_17929_MOESM2_ESM.pdf]

nature research

Corresponding author(s): *Saida Hadjab*

Last updated by author(s): 06 07 2020

Reporting Summary

Nature Research wishes to improve the reproducibility of the work that we publish. This form provides structure for consistency and transparency in reporting. For further information on Nature Research policies, see Authors & Referees and the Editorial Policy Checklist.

Statistics

For all statistical analyses, confirm that the following items are present in the figure legend, table legend, main text, or Methods section.

n/a	Confirmed	
☐	☒	The exact sample size (*n*) for each experimental group/condition, given as a discrete number and unit of measurement
☐	☒	A statement on whether measurements were taken from distinct samples or whether the same sample was measured repeatedly
☐	☒	The statistical test(s) used AND whether they are one- or two-sided<br>*Only common tests should be described solely by name; describe more complex techniques in the Methods section.*
☐	☒	A description of all covariates tested
☐	☒	A description of any assumptions or corrections, such as tests of normality and adjustment for multiple comparisons
☐	☒	A full description of the statistical parameters including central tendency (e.g. means) or other basic estimates (e.g. regression coefficient) AND variation (e.g. standard deviation) or associated estimates of uncertainty (e.g. confidence intervals)
☐	☒	For null hypothesis testing, the test statistic (e.g. *F*, *t*, *r*) with confidence intervals, effect sizes, degrees of freedom and *P* value noted<br>*Give P values as exact values whenever suitable.*
☒	☐	For Bayesian analysis, information on the choice of priors and Markov chain Monte Carlo settings
☒	☐	For hierarchical and complex designs, identification of the appropriate level for tests and full reporting of outcomes
☐	☒	Estimates of effect sizes (e.g. Cohen's *d*, Pearson's *r*), indicating how they were calculated

Our web collection on statistics for biologists contains articles on many of the points above.

Software and code

Policy information about availability of computer code

Data collection	single-cell data was collected by the Eukaryotic Single-cell Genomics facility at Science for Life Laboratory in Stockholm, Sweden. fastq demultiplexing was performed using deindexer (https://github.com/ws6/deindexer), aligment with STAR_2.4.2a(https://github.com/STAR) , deindexer commit version 19c200f186d8d2a77babe2435477998ec30e839e
Data analysis	Data analysis were allowed by ImageJ software, GraphPad Prism 6.<br>Main scRNAseq data analysis was performed with R packages (R3.5) and Seurat package 2: pagoda2 (https://github.com/hms-dbmi/pagoda2) ELpigraph.R version from commit 761a48a52457b05c108d9a326f23079a23b79046<br>crestree version from commit ca3cb44b4a714f6a5a3528fb06107b95a1bde075. All codes for downstreamscRNAseq data preprocessing and pseudotime analysis are deposited under the form of notebooks on the following github repository: https://github.com/LouisFaure/sensoryfates_paper

Data

Policy information about availability of data

All manuscripts must include a data availability statement. This statement should provide the following information, where applicable:

- Accession codes, unique identifiers, or web links for publicly available datasets
- A list of figures that have associated raw data
- A description of any restrictions on data availability

Raw data is available GEO under accession GSE150150 under the following link : https://www.ncbi.nlm.nih.gov/geo/query/acc.cgi?acc=GSE150150.

For TF activity analyis, JASPAR2018 database was accessed using the dedicated bioconductor R package (https://bioconductor.org/packages/release/data/annotation/html/JASPAR2018.html)

List of figures that have associated raw data:
Figure 1a and 1c,
Figure 1i
Figure 2
Figure 3b-d
Figure 3c-h
Figure 5
Figure 6c and 6d
Figure 6f
Supplementary Figure 2a

Field-specific reporting

Please select the one below that is the best fit for your research. If you are not sure, read the appropriate sections before making your selection.

☒ Life sciences ☐ Behavioural & social sciences ☐ Ecological, evolutionary & environmental sciences

For a reference copy of the document with all sections, see nature.com/documents/nr-reporting-summary-flat.pdf

Life sciences study design

All studies must disclose on these points even when the disclosure is negative.

[illegible]ample size	4910 cells sequenced after QC filtering. Initial filtering from sequenced plates to remove low quality cells or empty wells was done asfollowing: From the transcript count matrix, cells having less that 5x104 transcripts, less than 2500 genes and more than 25% of proportionof ERCC reads were filtered out.
[illegible]ata exclusions	2245 cells kept during analysis by computationally filtering out clusters not related to neural crest and sensory lineages (using knownmarkers) from the count matrix
[illegible]eplication	All experiment were done in replicate. For scRNAseq, Each plates sequenced is the result of the pooling of at least 3 embryos. Trunk E9.5: 1 plate, Trunk E10.5: 3 plates, Cranial E12.5 dataset: 2plates, DRG E9.5: 2 plates, DRG E10.5: 4 plates, DRG E11.5: 1 plate, DRG E12.5: 1 plate
Randomization	No randomization was done as mouse embryos as the only condition was wild type. The process of cell extraction of pooled embryos and sequencing is performed in an automated way, and does not discriminate a particular cell type.
Blinding	No blinding was done as mouse embryos as the only condition was wild type. The process of cell extraction of pooled embryos and sequencing is performed in an automated way, and does not discriminate a particular cell type.

Reporting for specific materials, systems and methods

We require information from authors about some types of materials, experimental systems and methods used in many studies. Here, indicate whether each material, [illegible]stem or method listed is relevant to your study. If you are not sure if a list[illegible]n applies to your research, read the appropriate section before selecting a response.

[illegible]aterials & experimental systems

n/a	Involved in the study	
☐	☒	Antibodies
☒	☐	Eukaryotic cell lines
☒	☐	Palaeontology
☐	☒	Animals and other organisms
☒	☐	Human research participants
☒	☐	Clinical data

Methods

n/a	Involved in the study	
☒	☐	ChIP-seq
☐	☒	Flow cytometry
☒	☐	MRI-based neuroimaging

Antibodies

[illegible]ntibodies used	Primary antibodies used were: rabbit anti-RUNX3 (gift from Jessell TM), rabbit anti-RUNX1((gift from Jessell TM), goat anti-TRKA (1:400, R&D Systems AF1056), goat anti-TRKB (1:500, R&D Systems AF1494), rabbit anti-TRKC (1:1000, Cell Signaling 3376), mouse anti-ISL1 (1:250, Developmental Studies Hybridoma Bank 39.4D5), goat anti-RET (1:100, R&D Systems AF482), goat anti-TRKC (1:500, R&D Systems AF1404), mouse anti-βIII-tubulin (1:1000, Promega G712A), chicken anti-RFP (1:250, Rockland 600-901-379S), mouse anti-NF200 (Sigma, N0142; 1:500), mouse anti-SOX10 (1:1000, Santa-Cruz, #sc-36569). After washing with PBS, Alexa Fluor secondary antibodies (Live Technology; 1:500)
Validation	Antibodies were previously used and validated in previous published studies, Lallemend et al. 2012 (EMBO journal), Hadjab/Franck et al,. 2013 (J. Neuroscience), Bartesaghi et al. 2018 (Cell reports) Wang/Wu et al., 2019 (Nat. Comms) Wang et al., 2019 (Development)

Eukaryotic cell lines

Policy information about cell lines

Cell line source(s)	*State the source of each cell line used.*
Authentication	*Describe the authentication procedures for each cell line used OR declare that none of the cell lines used were authenticated.*
Mycoplasma contamination	*Confirm that all cell lines tested negative for mycoplasma contamination OR describe the results of the testing for mycoplasma contamination OR declare that the cell lines were not tested for mycoplasma contamination.*
Commonly misidentified lines (See ICLAC register)	*Name any commonly misidentified cell lines used in the study and provide a rationale for their use.*

Palaeontology

Specimen provenance

Provide provenance information for specimens and describe permits that were obtained for the work (including the name of the issuing authority, the date of issue, and any identifying information).

Specimen deposition

Indicate where the specimens have been deposited to permit free access by other researchers.

Dating methods

If new dates are provided, describe how they were obtained (e.g. collection, storage, sample pretreatment and measurement), where they were obtained (i.e. lab name), the calibration program and the protocol for quality assurance OR state that no new dates are provided.

☐ Tick this box to confirm that the raw and calibrated dates are available in the paper or in Supplementary Information.

Animals and other organisms

Policy information about studies involving animals; ARRIVE guidelines recommended for reporting animal research

[illegible]boratory animals

Animals of either sex were included in this study. Animals were group-housed, with food and water ad libitum, under 12 h light–dark cycle conditions, under standardized condition in the animal facility of Karolinska Institutet.

[illegible]ild animals

No wild animals were used in this study

[illegible]eld-collected samples

No field-collected animals were used inthis study

[illegible]hics oversight

All animal work was performed in accordance with the national guidelines and approved by the local ethics committee of Stockholm, Stockholms Norra djurförsöksetiska nämnd.

Human research participants

Policy information about studies involving human research participants

Population characteristics

Describe the covariate-relevant population characteristics of the human research participants (e.g. age, gender, genotypic information, past and current diagnosis and treatment categories). If you filled out the behavioural & social sciences study design questions and have nothing to add here, write "See above."

Recruitment

Describe how participants were recruited. Outline any potential self-selection bias or other biases that may be present and how these are likely to impact results.

Ethics oversight

Identify the organization(s) that approved the study protocol.

Note that full information on the approval of the study protocol must also be provided in the manuscript.

Clinical data

Policy information about clinical studies

All manuscripts should comply with the ICMJE guidelines for publication of clinical research and a completed CONSORT checklist must be included with all submissions.

Clinical trial registration

Provide the trial registration number from ClinicalTrials.gov or an equivalent agency.

Study protocol

Note where the full trial protocol can be accessed OR if not available, explain why.

Data collection

Describe the settings and locales of data collection, noting the time periods of recruitment and data collection.

Outcomes

Describe how you pre-defined primary and secondary outcome measures and how you assessed these measures.

ChIP-seq

Data deposition

☐ Confirm that both raw and final processed data have been deposited in a public database such as GEO.

☐ Confirm that you have deposited or provided access to graph files (e.g. BED files) for the called peaks.

Data access links
May remain private before publication.

For "Initial submission" or "Revised version" documents, provide reviewer access links. For your "Final submission" document, provide a link to the deposited data.

Files in database submission | *Provide a list of all files available in the database submission.*

Genome browser session (e.g. UCSC) | *Provide a link to an anonymized genome browser session for "Initial submission" and "Revised version" documents only, to enable peer review. Write "no longer applicable" for "Final submission" documents.*

Methodology

Replicates | *Describe the experimental replicates, specifying number, type and replicate agreement.*

Sequencing depth | *Describe the sequencing depth for each experiment, providing the total number of reads, uniquely mapped reads, length of reads and whether they were paired- or single-end.*

Antibodies | *Describe the antibodies used for the ChIP-seq experiments; as applicable, provide supplier name, catalog number, clone name, and lot number.*

Peak calling parameters | *Specify the command line program and parameters used for read mapping and peak calling, including the ChIP, control and index files used.*

Data quality | *Describe the methods used to ensure data quality in full detail, including how many peaks are at FDR 5% and above 5-fold enrichment.*

Software | *Describe the software used to collect and analyze the ChIP-seq data. For custom code that has been deposited into a community repository, provide accession details.*

Flow Cytometry

Plots

Confirm that:

[x] The axis labels state the marker and fluorochrome used (e.g. CD4-FITC).

[x] The axis scales are clearly visible. Include numbers along axes only for bottom left plot of group (a 'group' is an analysis of identical markers).

[x] All plots are contour plots with outliers or pseudocolor plots.

[x] A numerical value for number of cells or percentage (with statistics) is provided.

Methodology

Sample preparation | DRG cells are disscociated, please see detailed in Methods section in FACS paragrah

Instrument | BD instruments. BD FACSDiva 8.0.2 and BD Influx were used.

Software | FlowJo v10 (BD) and BD FACS Sortware 1.2.0/142/Utopex 1.2.0.108

Cell population abundance | The cell population abundance is provide in Supplementary Figure 2. The initial number of events ranges between from 15653 to 50000, the percentage of tdtom+positive cells ranges from 0.2 to 8.81% of the total events. The plots presented in Supplementary Figure 2 are real data but represented one of many FACS experiments for the concerned mouse line.

Gating strategy | Gating strategy is shown in the Supplementary Fig. 2. Debris and erythrocytes were gated out from the total events using FSC-A versus SSC-A plotting. Doublets were further gated out using FSC-A versus FSC-H plotting. Finally, we plotted FSC-A versus PE-A (corresponding to TOMATO+ signal) to select cells that were TOMATO+ (a, b and e) or we plotted the FSC versus the 585/29 [561]-tdtomato (corresponding to TOMATO+ signal, c,d). A cell preparation negative for TOMATO was used to define the negative population (e). The BD Influx instrument was manually set up and calibrated on daily basis using BD CST and BD FACS *Accudrop beads.*

[x] a figure exemplifying the gating strategy is provided

Magnetic resonance imaging

Experimental design

Design type | *Indicate task or resting state; event-related or block design.*

Design specifications | *Specify the number of blocks, trials or experimental units per session and/or subject, and specify the length of each trial or block (if trials are blocked) and interval between trials.*

Behavioral performance measures | *State number and/or type of variables recorded (e.g. correct button press, response time) and what statistics were used to establish that the subjects were performing the task as expected (e.g. mean, range, and/or standard deviation across subjects).*

Acquisition

Imaging type(s)	*Specify: functional, structural, diffusion, perfusion.*
Field strength	*Specify in Tesla*
Sequence & imaging parameters	*Specify the pulse sequence type (gradient echo, spin echo, etc.), imaging type (EPI, spiral, etc.), field of view, matrix size, slice thickness, orientation and TE/TR/flip angle.*
Area of acquisition	*State whether a whole brain scan was used OR define the area of acquisition, describing how the region was determined.*

Diffusion MRI ☐ Used ☐ Not used

Preprocessing

Preprocessing software	*Provide detail on software version and revision number and on specific parameters (model/functions, brain extraction, segmentation, smoothing kernel size, etc.).*
Normalization	*If data were normalized/standardized, describe the approach(es): specify linear or non-linear and define image types used for transformation OR indicate that data were not normalized and explain rationale for lack of normalization.*
Normalization template	*Describe the template used for normalization/transformation, specifying subject space or group standardized space (e.g. original Talairach, MNI305, ICBM152) OR indicate that the data were not normalized.*
Noise and artifact removal	*Describe your procedure(s) for artifact and structured noise removal, specifying motion parameters, tissue signals and physiological signals (heart rate, respiration).*
Volume censoring	*Define your software and/or method and criteria for volume censoring, and state the extent of such censoring.*

Statistical modeling & inference

Model type and settings	*Specify type (mass univariate, multivariate, RSA, predictive, etc.) and describe essential details of the model at the first and second levels (e.g. fixed, random or mixed effects; drift or auto-correlation).*
Effect(s) tested	*Define precise effect in terms of the task or stimulus conditions instead of psychological concepts and indicate whether ANOVA or factorial designs were used.*

Specify type of analysis: ☐ Whole brain ☐ ROI-based ☐ Both

Statistic type for inference (See Eklund et al. 2016)	*Specify voxel-wise or cluster-wise and report all relevant parameters for cluster-wise methods.*
Correction	*Describe the type of correction and how it is obtained for multiple comparisons (e.g. FWE, FDR, permutation or Monte Carlo).*

Models & analysis

n/a	Involved in the study
☐	☐ Functional and/or effective connectivity
☐	☐ Graph analysis
☐	☐ Multivariate modeling or predictive analysis

Functional and/or effective connectivity	*Report the measures of dependence used and the model details (e.g. Pearson correlation, partial correlation, mutual information).*
Graph analysis	*Report the dependent variable and connectivity measure, specifying weighted graph or binarized graph, subject- or group-level, and the global and/or node summaries used (e.g. clustering coefficient, efficiency, etc.).*
Multivariate modeling and predictive analysis	*Specify independent variables, features extraction and dimension reduction, model, training and evaluation metrics.*
